# Fostering Friendship and Dating Skills Among Adults on the Autism Spectrum: A Randomized Controlled Trial of the Polish Version of the PEERS® for Young Adults Curriculum

**DOI:** 10.1007/s10803-023-05921-y

**Published:** 2023-04-12

**Authors:** Mateusz Płatos, Kinga Wojaczek, Elizabeth A. Laugeson

**Affiliations:** 1 Faculty of Psychology, University of Warsaw, Stawki 5/7, Warsaw, 00-183 Poland; 2 Association for Social Innovation “Mary and Max”, Marszałkowska 84/92/201, Warsaw, 00-514 Poland; 3 Department of Psychiatry and Biobehavioral Sciences, Semel Institute for Neuroscience and Human Behavior, University of California, 300 UCLA Medical Plaza, Los Angeles, CA 90095-6967 USA

**Keywords:** Autism Spectrum, Adulthood, Social Skills Training, Friendship, Dating, Telehealth Delivery

## Abstract

PEERS^®^ for Young Adults is an evidence-based program aimed at teaching social skills needed to establish and maintain close relationships, including friendship and romantic relationships. The study investigated the effects of the Polish adaptation of the curriculum on the social functioning of adults on the autism spectrum. Fifteen young adults (aged 18–32, *M* = 23.5) were randomly allocated to an immediate or delayed (control) treatment condition. By self and parent report, individuals who participated in the PEERS^®^ program showed significant improvements in social skills, social cognition, and social knowledge, but limited gains in social engagement. The effects were maintained over six months after the treatment. The intervention was well-accepted and deemed feasible by young adults, their parents, and peers involved in the program.

**Supplementary Information:**

The online version contains supplementary material available at 10.1007/s10803-023-05921-y.

Loneliness, social isolation, and lack of social support are known factors negatively impacting the quality of life in adults on the autism spectrum (Bishop-Fitzpatrick et al., 2018; Lin & Huang, 2019; Mason et al., 2018). Although most autistic people express interest in friendship and romantic relationships (Strunz et al., 2017), they may also struggle to form and sustain them. They report having fewer friends, less often having romantic relationships, and receiving less social support than non-autistic people (Bishop-Fitzpatrick et al., 2018). Moreover, autistic adults are less likely to have peer relationships compared to autistic adolescents (Orsmond et al., 2004). Initiating and maintaining social contacts may be more demanding in social environments typical for adults, such as the workplace or university, and for those who are not engaged in employment or education, opportunities for socializing are even more limited (Hancock et al., 2020).

One of the factors contributing to the social isolation of people on the autism spectrum is their social and communication difficulties (American Psychiatric Association, 2013; World Health Organization [WHO], 2022). In youth and adults, these difficulties include starting and holding a conversation, appropriate use of humor, use of social media and electronic communication, conflict resolution, and dating skills (Burke et al., 2010; Cola et al., 2022; Moody & Laugeson, 2020). Behaviors countering social norms and expectations, such as inappropriate courting, can lead to painful rejections and victimization (Brown-Lavoie et al., 2014; Stokes, Newton, & Kaur, 2007). Consequently, social skills deficits are linked to smaller social networks among autistic adults (Pallathra et al., 2018). Moreover, low social engagement may lead to increased anxiety in peer interactions and fewer opportunities to learn from others about social behavior (Hancock et al., 2020). Thus, social skills (and knowledge) and social engagement are interrelated and both are common targets of therapeutic intervention.

Social Skills Training (SST) is an evidence-based intervention based on direct instruction, modeling, and practicing social skills, often in a group setting. SST is one of the most common treatments for individuals on the autism spectrum (Ishler et al., 2021; Płatos & Pisula, 2019) and accumulated a large body of evidence (Hume et al., 2021). However, existing studies include mostly children and adolescents, while data on the interventions designed for adults is still limited.

A recent literature review revealed 18 group-based studies examining the efficacy of SST programs for adults on the autism spectrum, including eight randomized controlled trials (RCTs; Dubreucq, Haesebaert, Plasse, Dubreucq, & Franck, 2021). Of these RCTs, one compared SST with a non-training group facilitating social interactions, yielding no significant differences (Ashman et al., 2017), while three others focused on job-related skills (Gorenstein et al., 2020; Morgan, Leatzow, Clark, & Siller, 2014; Oswald et al., 2018). The remaining four studies examined the same, manualized intervention – the Program for the Education and Enrichment of Relational Skills for Young Adults (PEERS^®^ for Young Adults; Laugeson, 2017). These studies include two RCTs conducted by the authors of the intervention (Gantman et al., 2012; Laugeson, Gantman, Kapp, Orenski, & Ellingsen, 2015), one replication (McVey et al., 2016), and one secondary analysis (McVey et al., 2017). Together, they indicate that autistic young adults that participated in the PEERS^®^ program improved their social skills, social knowledge, empathy, and social engagement with peers, compared to delayed treatment control.

The above review reveals important gaps in the current literature. First, among the studies analyzed by Dubreucq and colleagues (2021), only one shows the maintenance of social skills acquired during the treatment over time (Laugeson et al., 2015). Yet, the durability of the intervention outcomes is one of the crucial indicators of its efficacy and clinical significance. Second, as pointed out by Monahan, Freedman, Pini, and Lloyd (2021), the majority of studies on the efficacy of SSTs do not gather any feedback from autistic individuals regarding the treatment relevance and satisfaction. Input from the autistic community is imperative for providing support that is seen as valid and meaningful by service users. Third, 88.8% of the studies reviewed by Dubreucq and colleagues (2021) were conducted in the USA. As social skills are culturally diverse, SSTs are also not universal but must be adapted to the social norms and expectations unique to each cultural group (Davenport et al., 2018). Unfortunately, there are scarce evidence-based SSTs for autistic adults outside the USA.

In their systematic review of cultural adaptations of SSTs designed for autistic people, Davenport and colleagues (2018) found only five studies that had described some cultural modifications of the social skills curriculums, but none of them included adult participants. Since then, a recent RCT by Oh and colleagues (2021) reported an adaptation and validation of the PEERS^®^ for Young Adults program in South Korea. However, results indicated immediate improvements only in social knowledge, with no gains in social skills or social engagement. Thus, the efficacy of the culturally adapted SSTs for autistic adults, including the PEERS^®^ program, warrants further investigation.

The main goal of the current study was to examine the efficacy and ecological validity of the culturally adapted version of the PEERS^®^ for Young Adults. It was hypothesized that young adults on the autism spectrum participating in the PEERS^®^ program would increase (a) their social knowledge, (b) social skills, in particular concerning peer interactions, (c) and social engagement with peers (the number of get-togethers with friends and the number of dates), as well as (d) their self-report level of empathy. It was predicted that these outcomes will maintain over time.

## Methods

### Participants

Participants were 15 young adults (73.3% males, all Caucasian/White) between 18 and 32 years old (*M* = 23.5, *SD* = 4.2) with a clinical diagnosis of Asperger’s Syndrome (*n* = 13) or childhood autism (*n* = 2), according to ICD-10 (WHO, 1992). Over forty-six percent (46.7%) reported co-existing psychiatric conditions, including depression, social phobia, obsessive disorder, and Tourette’s Syndrome, and 60% received psychotropic medication. The majority of young adults (86.7%) lived with their parents while two lived independently. They were mostly still receiving an education (60.0%) and the rest held a high school (*n* = 3) or university (*n* = 2) degree. One participant had a part-time job. Reporting parents were 13 mothers and 2 fathers, predominantly with a university degree (80.0%). Detailed demographic information on the treatment and control groups is reported in Table 1.

**Table 1:** Characteristics of participants at baseline (T1)

Variables	Treatment Group (*n* = 6)		Waitlist Control Group (*n* = 9)	
	*M (SD)*		*M (SD)*	*p*
Age (years)	22.6 (2.9)		24.04 (5.0)	ns
Gender (% male)	66.7		77.8	ns
ASD diagnosis (%)				ns
Asperger Syndome	83.3		88.9	
Childhood autism	16.7		11.1	
Comorbid psychiatric disorders (%)	33.3		55.6	ns
On medication (%)	50.0		66.7	ns
Education (%)				ns
Secondary education	16.7		11.1	
Post-secondary non-tertiary education	33.3		11.1	
Tertiary education	33.3		22.2	
Completed education	16.7		55.6	
*Baseline Measures*				
ADOS-2 Comparison Score	6.67 (1.37)		5.60 (0.55)	ns
Stanford-Binet 5 Matrices	10.67 (3.56)		11.80 (4.09)	ns
Stanford-Binet 5 Vocabulary	10.00 (4.10)		11.40 (2.30)	ns
Stanford-Binet 5 Total IQ	101.00 (23.18)		107.60 (9.02)	ns
ABAS-3 Communication	54.67 (8.94)		59.25 (8.96)	ns
ABAS-3 Social	45.17 (9.22)		53.13 (7.00)	ns
ABAS-3 Self-direction	49.33 (13.11)		56.25 (9.59)	ns
*Young Adult Report Outcome Measures*				
TYASSK	15.00 (2.83)		15.79 (1.47)	ns
QSQ Total get-togethers	2.00 (2.68)		2.46 (1.95)	ns
EQ	27.67 (6.28)		27.03 (11.43)	ns
*Parent Report Outcome Measures*				
QSQ Total get-togethers	1.83 (2.56)		4.71 (4.82)	ns
SRS-2 Total	103.67 (17.84)		90.10 (28.79)	ns
ASRS Total	110.50 (26.76)		92.54 (30.24)	ns
ASRS Peer Socialization	21.67 (2.73)		16.90 (4.27)	ns

Note. ns = statistically non-significant. TYASSK = Test of Young Adult Social Skills Knowledge; QSQ = Quality of Socialization Questionnaire; SRS-2 = Social Responsiveness Scale-2; ASRS = Autism Spectrum Rating Scale; EQ = Empathy Quotient

Participants were recruited through social media, local organizations offering services for adults on the autism spectrum or referrals by mental health professionals. To be eligible for the treatment young adults had to satisfy the following criteria: (a) age between 18 and 35 years of age; (b) a previous diagnosis of Pervasive Developmental Disorder (including Asperger Syndrome and childhood autism), according to ICD-10 (WHO, 1992); (c) absence of co-occurring intellectual disability (IQ > 70); (d) absence of a major, concurrent psychiatric disorder (e.g., schizophrenia, bipolar disorder); (e) absence of oppositional/aggressive behavior; (f) occurrence of difficulties in developing and/or maintaining friendship, romantic relationships, or other peer relations; (g) motivation to participate in the treatment; and (h) consent to take part in all the assessments, including recording of the treatment sessions.

The screening process consisted of three stages. First, young adults or their parents who filled out a short application form on the program’s website were screened via phone interview to verify formal criteria (age and diagnosis) and time availability. The availability of a family member as a social coach was also discussed, but it was not a criterion to participate in the trial. Second, a young adult was invited for an intake interview with a psychologist who evaluated their mental health status, peer problems, and motivation for treatment. A parent was invited for the interview if they were willing to serve as a social coach. Third, standardized assessments were conducted using the Autism Diagnostic Observation Schedule, Second Edition (ADOS-2; module 4; Chojnicka and Pisula, 2017) to confirm the autism spectrum diagnosis, the Abbreviated Battery of the Stanford-Binet Intelligence Scales, Fifth Edition (Sajewicz-Radtke et al., 2017) to assess intellectual functioning, and the Adaptive Behavior Assessment System, Third Edition (ABAS-3; Otrębski, Domagała-Zyśk, & Sudoł, 2019) to measure adaptive skills.

**Fig. 1:**
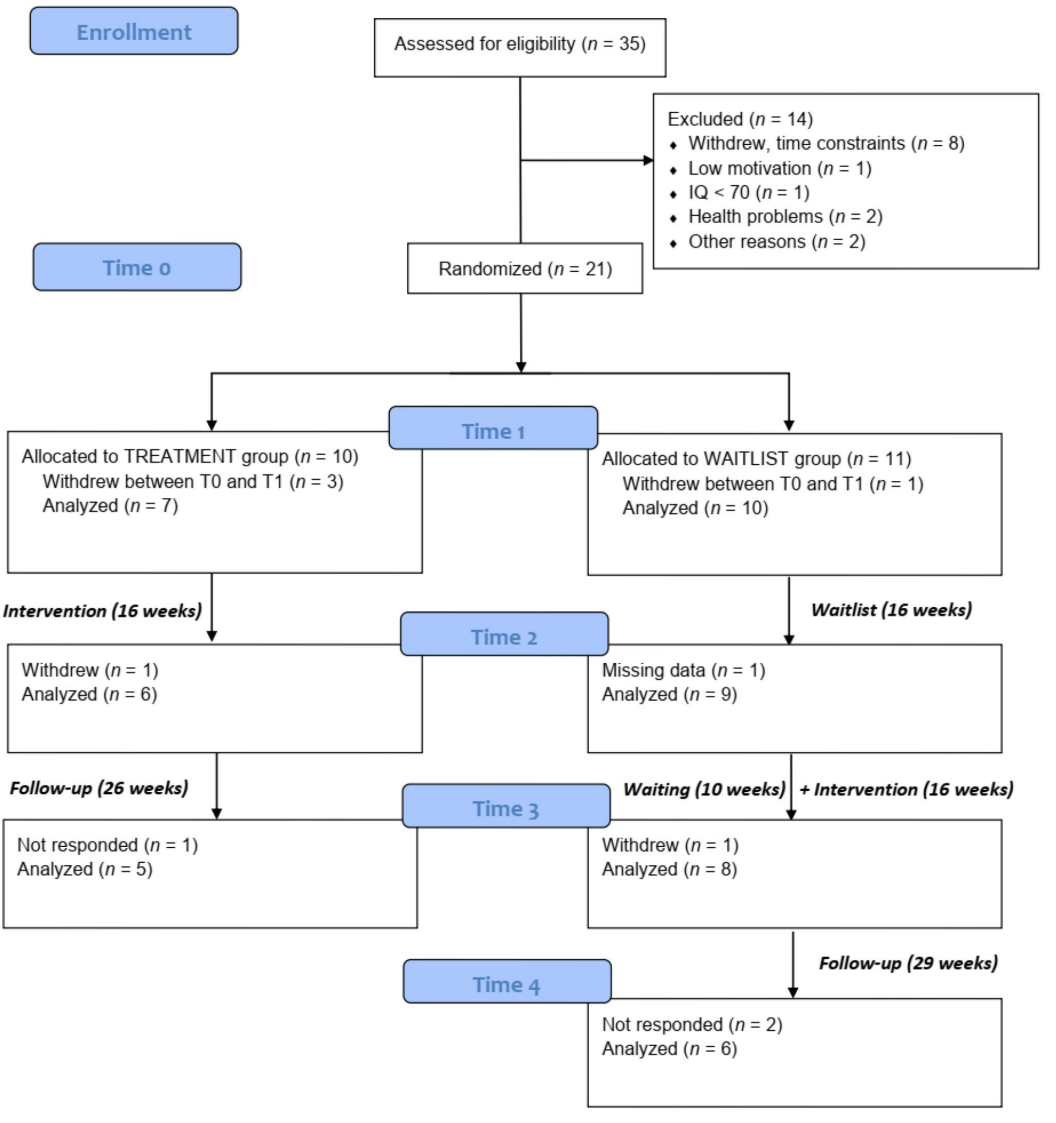
CONSORT flow diagram

Thirty-five young adults were screened for eligibility, of which 21 were randomized into a Treatment Group (TG; *n* = 10) and a Waitlist Control Group (WCG; *n* = 11). Four participants withdrew during the pandemic-related waiting period before the commencement of the trial (see “Study design and procedures”). One participant in TG withdrew during the treatment, citing discomfort with the transition to remote mode as the reason (see below), and data from one participant in WCG was missing. The final sample, used for the main analyses, consisted of six participants in TG and nine participants in WCG. One further participant withdrew from WCG during a delayed treatment due to elevated depressive symptoms. The full flow of study subjects is shown in Fig. 1.

### Study design and procedures

Participants enrolled in the study were randomly assigned to TG or WCG using permuted block randomization (with block sizes of 2 or 4) performed via web-based software (Sealed Envelope Ltd, 2022). Randomization was conducted by an independent allocator who was not related to the study and knew only participants’ unique ID and gender. That latter information was used to avoid the situation with only one identifying male or female in a treatment group, as recommended by the program’s manual (Laugeson, 2017). The group allocation was revealed to participants and research staff after baseline measurement was completed (T0).

The start of the intervention phase of the study was set for March 2020, but the outburst of the COVID-19 pandemic and the introduction of mobility restrictions by the Polish government forced postponing of the intervention by six months. To control for the possible imbalance between the study arms that could appear during the waiting period, the baseline measurement was repeated before the intervention started (T1). Both groups were assessed again after 16-weeks when TG completed the intervention (T2). Before WCG received the treatment, there was another waiting period (10-weeks) related to the COVID-19 pandemic. After WCG completed the intervention, both groups were assessed one more time (T3: follow-up for TG; post-test for WCG). Lastly, outcome measures were collected 29-weeks after WCG completed the treatment to examine the maintenance of its effects (T4: follow-up for WCG). Figure 1 shows a CONSORT diagram of the study.

Further changes to the study protocol were made as a result of the pandemic-related restrictions on social gatherings and mobility. In particular, the peak of the second wave of the coronavirus in November 2020 forced the authors to move the last seven (out of 16) sessions of TG to an online setting. All the program components, including group discussion, role-play demonstrations, and behavioral rehearsal exercises, were recreated using synchronous online tools (Zoom Meetings software). In WCG, all the sessions were held in person, as the mobility restrictions were loosened. However, participants in both conditions could socialize online and with other PEERS^®^ group members as a part of their weekly assignments, which is usually not recommended in the PEERS^®^ program (Laugeson, 2017).

Therapeutic personnel of the program consisted of two group leaders (one for the young adult groups and one for the social coach groups) and two behavioral coaches assisting in role-play demonstrations, behavioral rehearsal exercises, and behavior management in young adult groups. Groups leaders were psychologists with extensive experience in supporting autistic adults. The group leader working with young adults was a PEERS^®^ for Young Adults Certified Provider who had previously served as an intern at the UCLA PEERS^®^ Clinic under the supervision of the program’s author. The second group leader was trained in the PEERS^®^ curriculum during the program’s pilot. Behavioral coaches were either psychologists or psychology students working under the group leader’s supervision.

### Overview of PEERS® and cultural adaptations

The PEERS^®^ for Young Adults curriculum is a manualized social skills training designed for adults on the autism spectrum (Laugeson, 2017). The program consists of sixteen 90-minute sessions held once a week in a group of 6–12 participants, typically led by mental health professional or educator. The curriculum covers social skills relevant to initiating and maintaining peer relations, particularly friendship and romantic relationships, such as starting and holding a conversation, appropriate use of humor, organizing get-togethers with friends, resolving conflicts, starting and ending a phone call, using social media, and dating (see Table S1 in the Appendix for a complete list of topics).

Teaching methods are based on cognitive-behavioral principles, including direct instruction of relevant skills, role-play demonstrations of target behaviors, perspective-taking questions, and behavioral rehearsal exercises. After each session, participants receive homework, involving practicing the skills and socializing with peers (both in-group and out-group) to foster generalization. Young adults are supported by a parent or another adult, called a ‘social coach’ in the program. Social coaches participate in parallel sessions, in which they are taught the same didactic material and learn how to facilitate young adults’ skills acquisition and usage. Between the sessions, social coaches practice new skills with young adults and support homework completion, thereby enhancing treatment compliance and skills generalization.

The adaptation process consisted of three main stages: (a) linguistic translation and initial adaptation of program materials, (b) a pilot of the intervention, and (c) refinement of the curriculum and preparation of the final version for the randomized controlled trial.

Although the original intervention manual was published recently (2017) and remained the primary source of the adaptation, it was further updated by the materials from the UCLA PEERS^®^ Certified Training Seminar (2018), and the Telehealth version of PEERS^®^ shared by its author. Session synopses and all the auxiliary materials (e.g., screening interview forms, handouts for participants, and social coaches) were translated by the first author. Subsequently, the first and the second author held a 3-hour meeting on each of the 16-sessions, discussing the content that needed to be adapted. Moreover, about 100 role-play videos demonstrating the target skills were remade with adjusted scenarios. Changes included language considerations (e.g. different meaning of a ‘friend’ in the Polish context), conversational conventions, as well as popular peer groups/crowds, jokes, and teasing comebacks. A full list of the adaptations can be found in Table S1 in the Appendix.

A pilot of the intervention was a pre-post comparison (without a control group) of eight young adults (*M*_*age*_ = 20.2; *SD* = 2.1; range = 18–24; all males) that took part in the first group of the adapted PEERS^®^ curriculum. The group was led by the first author who concurrently trained the staff that served as group leaders and facilitators during the main trial. The pilot showed statistically significant results or positive trends in the outcome variables, indicating adequate sensitivity of the measures and feasibility of the adapted intervention.

Several refinements to the program were made as a result of the pilot. First, it was concluded that parents were not always best matches as social coaches for young adults, both for organizational reasons (living in another city, scheduling conflicts, or lack of time) and psychological ones (parental burn-out, tension or conflict in a parent-young adult relationship, parent’s personal problems). Other relatives were often unavailable for similar reasons. Thus, the research team decided to include trained psychology students in the treatment (as part of their internship) when parents were unavailable or unsuitable for social coaching. Peer mediation is used regularly by the intervention developer and was already tested during the Polish pilot, turning out to be successful in the case of one participant who did not have an available relative. Moreover, peer mediation was deemed appropriate and sometimes preferable for young adults, particularly when practicing and discussing topics related to dating that might be embarrassing to review with one’s parents. Consequently, in the main study, two peer coaches were included in TG (including one that supported a participant who withdrew during the intervention) and seven in WCG (vs. five and two parents in these groups, respectively).

Second, some program materials were modified to address participants’ and social coaches’ needs. Specifically, homework assignment worksheets, in which social coaches report their homework completion, were adjusted from open-ended to checklist form, as the latter turned out to be easier to follow. Similar worksheets were prepared for young adults to provide them with more responsibility and control in homework completion.

Third, specific terms that were unclear for young adults in the pilot were modified. Moreover, after consulting the curriculum’s premises with autistic self-advocates, the generic name of the intervention was changed from ‘social skills training’ to ‘social skills workshop’, because the term ‘training’ had negative connotations for some individuals.

### Outcome Measures

#### Test of Young Adult Social Skills Knowledge (TYASSK; Laugeson, 2017)

TYASSK is a criterion-based measure of knowledge about social skills, prepared specifically to assess the efficacy of PEERS^®^ for Young Adults. It consists of 30 questions, each with two answers, of which one is correct (for example, “When you first start dating someone, you should: a. Tell them about your dating history; b. Avoid talking about your dating history”; b. is correct). The measure was translated and adapted to Polish for the current trial. The original scale proved to be sensitive to changes in social skills knowledge in the previous studies (Gantman et al., 2012; Laugeson et al., 2015), with the Korean adaptation of the TYASSK showing similar effects (Oh et al., 2021). The measure internal consistency was very poor at baseline (Cronbach’s α = 0.19), reflecting participants’ lack of knowledge of the correct responses and picking up the answers randomly (corroborated also by the mean expected at random: *M* = 15.47 in 0–30 range with dichotomous coding). In contrast, after both groups completed the treatment (T3), the Cronbach’s α was acceptable at the level of 0.72.

#### Quality of Socialization Questionnaire (QSQ for Young Adults; Laugeson, 2017)

The QSQ is a self-report and parent-report measure of young adults’ social engagement with peers. Respondents are asked about the frequency of social activities in the previous month outside organized extracurriculars, namely: (a) the number of get-togethers that young adults organized, (b) the number of get-togethers they were invited to, (c) the number of dates they organized, and (d) the number of dates they were invited to. Participants were instructed not to include meetings with other PEERS^®^ group members. In order to validate the answers, respondents were also asked to provide a list of names of peers that the young adult had met. As the trial was conducted under pandemic-related mobility restrictions, virtual get-togethers that used synchronous communication tools could be counted in the total number of social activities. To avoid inflating the number of outcome variables, the numbers of hosted and invited events were combined into single variables (i.e. the total number of get-togethers and the total number of dates). The measure was translated to Polish for use in the current trial. It was previously used to assess the outcomes of the original PEERS^®^ curriculum (Gantman et al., 2012; Laugeson et al., 2015), as well as its Korean adaptation (Oh et al., 2021).

#### Social Responsiveness Scale-2 (SRS-2; Constantino & Gruber, 2012)

The Social Responsiveness Scale-2 (SRS-2) is a screening questionnaire measuring social impairment related to autism, completed by adults or their relatives. It consists of 65 statements rated on a 4-point Likert scale, from “not at all” to “almost always”. The higher Total Score indicates more difficulties. The scale also includes five treatment subscales: Social Awareness, Social Cognition, Social Communication, Social Motivation, and Restricted Interests and Repetitive Behavior. The Polish version of the measure was prepared for the current study. First, it was translated by the first author and then translated back to English by an independent translator. The authors of the original scale compared and commented on the differences between the versions. This process was repeated until full concordance was achieved. Parent report was used in this study, showing very good internal consistency (Cronbach’s α = 0.93).

#### Autism Spectrum Rating Scales (ASRS; Goldstein & Naglieri, 2010)

The Autism Spectrum Rating Scales (ASRS) is another screening questionnaire designed to measure behaviors associated with the autism spectrum. It consists of 71 items rated on a 5-point Likert scale from “Never” to “Very often”. The scale includes the Total Score and several subscales, of which Peer Socialization was chosen for the current study, as it indicates problems in interactions with peers. The questionnaire was adapted to Polish by E. Wrocławska-Warhała and R. Wujcik (2016) showing high reliability (Cronbach’s α > 0.8) and validity. However, it contains norms only for children and adolescents up to 18 years of age, so raw scores were used in this study to examine the treatment effects. In the present sample, the scale showed excellent internal consistency (Cronbach’s α = 0.95).

#### Empathy Quotient (EQ; Baron-Cohen & Wheelwright, 2004)

The Empathy Quotient (EQ) is a self-report measure of empathy, including both a cognitive domain (i.e. the ability to understand or predict mental states of other people; social cognition) and affective domain (i.e. the ability to react emotionally to other people’s emotions). Although a three-factor structure of the scale has also been proposed (cognitive empathy, emotional reactivity, and social skills; Muncer & Ling, 2006), psychometric analyses confirmed the unidimensional character of the measure (Allison et al., 2011). The questionnaire consists of 40 items using a 4-point Likert scale, from ‘strongly disagree’ to ‘strongly agree’. A higher score (range 0–80) indicates a higher level of empathy. The measure was used in the original studies of the PEERS^®^ for Young Adults curriculum (Gantman et al., 2012; Laugeson et al., 2015). It was translated to Polish by Agnieszka E. Wainaina-Wozna. In the current study, the measure showed acceptable reliability (Cronbach’s α = 0.83).

### Ecological Validity

To elicit feedback on the treatment acceptability and satisfaction, young adults and social coaches (including parents and peers) were asked seven close-ended questions regarding: (a) helpfulness of the main program components, (b) time burden to participate in the program for young adults and social coaches, (c) overall impact of the curriculum on skills related to initiating and maintaining close relationships, and (d) willingness to recommend the program to others. The answers were provided on the 7-point Likert scales (e.g., from 1 - not helpful at all; 7 - very helpful; see Table S2 and Table S3 in the Appendix for the list of questions).

Moreover, young adults and social coaches were asked two open-ended questions: “What did you like about the program?” and “What would you change or add to the program?” Additionally, peers serving as social coaches were asked: “What did you learn from participating in the PEERS^®^ internship program?” The answers were examined using thematic analysis conducted in Atlas.ti 9 software.

### Treatment Fidelity

To ensure the treatment’s compliance with the adapted PEERS^®^ manual, all the sessions with young adults and social coaches were recorded and monitored by the first author who is a PEERS^®^ Certified Provider. Participants’ attendance and weekly homework completion rates were also collected. In TG, young adults attended, on average, 14.8 out of 16 sessions, while in WCG, they attended 15.4 sessions. Homework completion was high. For example, for having an in-group phone call to practice trading information (six times during the program with different participants) the completion rate was 100% (data for TG). Another important homework assignment, introduced due to pandemic restrictions, was to have an online meeting with another group member (a longer video chat involving a conversation and some activities), for which the completion rate was also 100%. However, for organizing a get-together with a friend (online or in-person) the completion was only 44.4%, presumably reflecting limited opportunities for socialization during a lockdown.

### Data Analysis

To verify the study’s hypotheses, mixed analyses of variance (ANOVAs) were performed (Group x Time). Significant interaction effects were indicating the conditions’ differential impact on outcome variables. The main analyses were followed up by simple effects analyses to examine the direction of change. Because of the sensitivity of the ANOVA’s assumption of homogeneity of variance-covariance matrices, the level of significance for the Box’s M Test was set to 0.001 (Verma, 2015). The Benjamini-Hochberg procedure was used to avoid inflating type I error due to multiple comparisons (Benjamini & Hochberg, 1995). The False Discovery Rate (FDR) was set at 0.10. All the statistical analyses were performed with IBM SPSS 27 software.

## Results

### Preliminary Analyses

The data was screened for potential outliers. For primary analyses, six outliers were found out of 194 data points (3.09%) – three in SRS-2 Total Score, two in young adult-reported QSQ, and one in parent-reported QSQ. In the case of QSQ, values were compared with the list of the peers’ names provided by participants in the questionnaire to assess their credibility. As a result, four data points remained in the analyses and two were adjusted using the winsorization procedure (Wilcox, 2005). All the statistical analyses were run with adjusted and unadjusted values, yielding comparable results.

There were few item-level missing values (< 1.5% of items for each scale), which were missing completely at random, according to Little’s MCAR test (Little, 1998). These data points were computed using the Expectation-Maximization procedure (Dempster et al., 1977). Furthermore, one young adult report and one parent report were missing at T1 (repeated baseline) but present at T0 (primary baseline). Due to the high stability of the baseline measurements (see below), data from T0 was used in these two cases, corrected by a mean difference between T1 and T0 on each scale. One young adult report and two parent reports were missing at T2, so they were excluded from the analysis.

Only five young adults and three parents reported young adults’ dates at any time point in QSQ, so this variable was excluded from further analyses.

### Conditions Comparability

Chi-square and t-test were used to examine potential differences in demographic characteristics between TG and WCG at baseline (T0), yielding no significant results. Similarly, there were no differences in autism severity (ADOS-2), intelligence scores (Stanford-Binet 5), or adaptive skills (ABAS-3). Finally, no significant differences were found among outcome measures at baseline (T0).

To assess differences in outcome measures between the first (T0) and second (T1) baseline, a within-subjects t-test was used. Outcome variables showed high stability, as no statistically significant differences were found (*p* < .05). The ASRS Peer Socialization score decreased over time (= fewer difficulties) at the tendency level (*p* = .050).

Comparisons of the outcome measures between TG and WCG were repeated for the second baseline (T1), yielding no significant differences, except for the ASRS Peer Socialization subscale, in which TG showed higher scores (= more difficulties) than WCG (*t*(12) = 2.38; *p* = .035).

The majority of participants (66.7%) did not receive any other behavioral treatment during the trial. In TG, two young adults attended individual psychotherapy. In WCG, two young adults attended individual and group therapy, and one participant attended individual therapy only. Fifty percent and 66.7% of participants received psychotropic medication in TG and WCG, respectively. There were no significant differences in the rates of either behavioral or pharmacological therapy between the groups.

### Treatment Efficacy

To verify the study’s main hypotheses, mixed analyses of variance (ANOVA) with Time as a within-subjects factor (T1 vs. T2) and Group as a between-subjects factor (TG vs. WCG) were conducted. Analyses showed significant interaction effects (Group x Time) in four out of six variables that met the requirements for a mixed ANOVA: self-reported knowledge about social skills (TYASSK), parent-reported level of autism-related difficulties (SRS-2 and ASRS total scores), and the quality of socialization with peers (ASRS Peer Socialization). All the effects were large (η^2^ > 0.14) and remained significant after applying a correction for multiple comparisons, as shown in Table 2.

**Table 2:** Means, standard deviations, and mixed ANOVAs results for treatment and waitlist control groups

Outcome variables	Treatment (*n* = 6)				Control (*n* = 9)					
	Pre (T1)		Post (T2)		Pre (T1)		Post (T2)			
	*M (SD)*		*M (SD)*		*M (SD)*		*M (SD)*	Time x Group	*p*	*η*_*p*_^*2*^
*Young adult report*										
TYASSK	15.00 (2.83)		22.00 (4.05)		15.79 (1.47)		15.89 (2.03)	29.01	< 0.001*	0.691
QSQ Total get-togethers	2.00 (2.68)		3.17 (2.64)		2.46 (1.95)		3.89 (2.80)	1.03	0.857	0.003
EQ	27.67 (6.28)		31.50 (13.05)		27.03 (11.43)		25.67 (8.89)	1.72	0.212	0.117
*Parent report*										
SRS-2 Total Score^a^	103.40 (19.93)		77.20 (16.80)		90.10 (28.79)		88.11 (26.81)	17.02	0.001*	0.586
ASRS Total Score^b^	108.80 (29.55)		75.00 (24.36)		92.54 (30.24)		100.38 (35.39)	113.33	< 0.001*	0.912
ASRS Peer Socialization^b^	21.40 (2.97)		16.00 (5.20)		16.90 (4.27)		19.12 (3.44)	29.22	< 0.001*	0.726
QSQ Total get-togethers^c^	0.80 (0.45)		4.40 (2.07)		4.71 (4.82)		4.00 (4.12)	N/A^c^	N/A^c^	N/A^c^

Note. TYASSK = Test of Young Adult Social Skills Knowledge; QSQ = Quality of Socialization Questionnaire; SRS-2 = Social Responsiveness Scale-2; ASRS = Autism Spectrum Rating Scale; QSQ = Quality of Socialization Questionnaire; EQ = Empathy Quotient

^a^ n = 14; ^b^ n = 13; ^c^ n = 12, non-parametric test results reported in the text

* Statistically significant under Benjamini-Hochberg procedure (FDR = 0.10).

Post-hoc simple effects analyses using univariate, within-subjects ANOVAs were conducted to explore the nature of the interaction effects. Young adults who participated in the PEERS^®^ curriculum increased their knowledge about social skills (*F* (1, 5) = 21.62; *p* = .006; partial η^2^ = 0.812), while those waiting for the intervention did not (*F* (1, 8) = 0.86; *p* = .777; partial η^2^ = 0.011). Moreover, in TG, parents of autistic young adults reported a decrease of autism-related difficulties as measured by the SRS-2 (*F* (1, 4) = 37.84; *p* < .004; partial η^2^ = 0.904), while in WCG, there was no change (*F* (1, 8) = 0.30; *p* = .601; partial η^2^ = 0.036). Exploratory analyses of the SRS-2 treatment subscales showed a significant improvement in TG for Social Cognition (*F* (1, 4) = 13.57; *p* = .021; partial η^2^ = 0.772), Social Communication (*F* (1, 4) = 18.71; *p* = .012; partial η^2^ = 0.824), Social Motivation (*F* (1, 4) = 8.30; *p* = .045; partial η^2^ = 0.675), and Restricted Interests and Repetitive Behavior change (*F* (1, 4) = 40.00; *p* = .003; partial η^2^ = 0.909), but not for Social Awareness (*F* (1, 4) = 0.77; *p* = .430; partial η^2^ = 0.161).

In TG, parents also noted a significant decrease of the core autism difficulties measured by ASRS (*F* (1, 4) = 121.02; *p* < .001 partial η^2^ = 0.968). In contrast, parents in WCG reported an increase in autism-related difficulties (*F* (1, 7) = 10.45; *p* = .014; partial η^2^ = 0.599). A similar pattern of results was obtained in Peer Socialization, the treatment subscale of ASRS. The level of difficulties in peer relations decreased in TG (*F* (1, 4) = 19.97; *p* = .011; partial η^2^ = 0.833) but increased in WCG (*F* (1, 7) = 7.28; *p* = .031; partial η^2^ = 0.510). All the effects remained statistically significant after adjustment for multiple comparisons using the Benjamini-Hochberg procedure (Benjamini & Hochberg, 1995).

Due to violation of requirements for ANOVA, the parent-reported number of young adults’ get-togethers with peers was analyzed using a non-parametrical test. The U Mann-Whitney test showed a statistically significant difference between TG and WCG on difference scores (*U* = 3.00; *Z* = -2.406; exact *p* = .018; Cohen’s *r* = .69). Median change between T1 and T2 in the number of get-togethers in the previous month was four for TG but zero for WCG.

The interaction effects for a self-reported number of get-togethers and empathy were not statistically significant, although the effects showed expected directions with small (QSQ Total get-togethers) or medium effect sizes (EQ; see Table 2). The main effects of Group and Time were not significant.

**Fig. 2:**
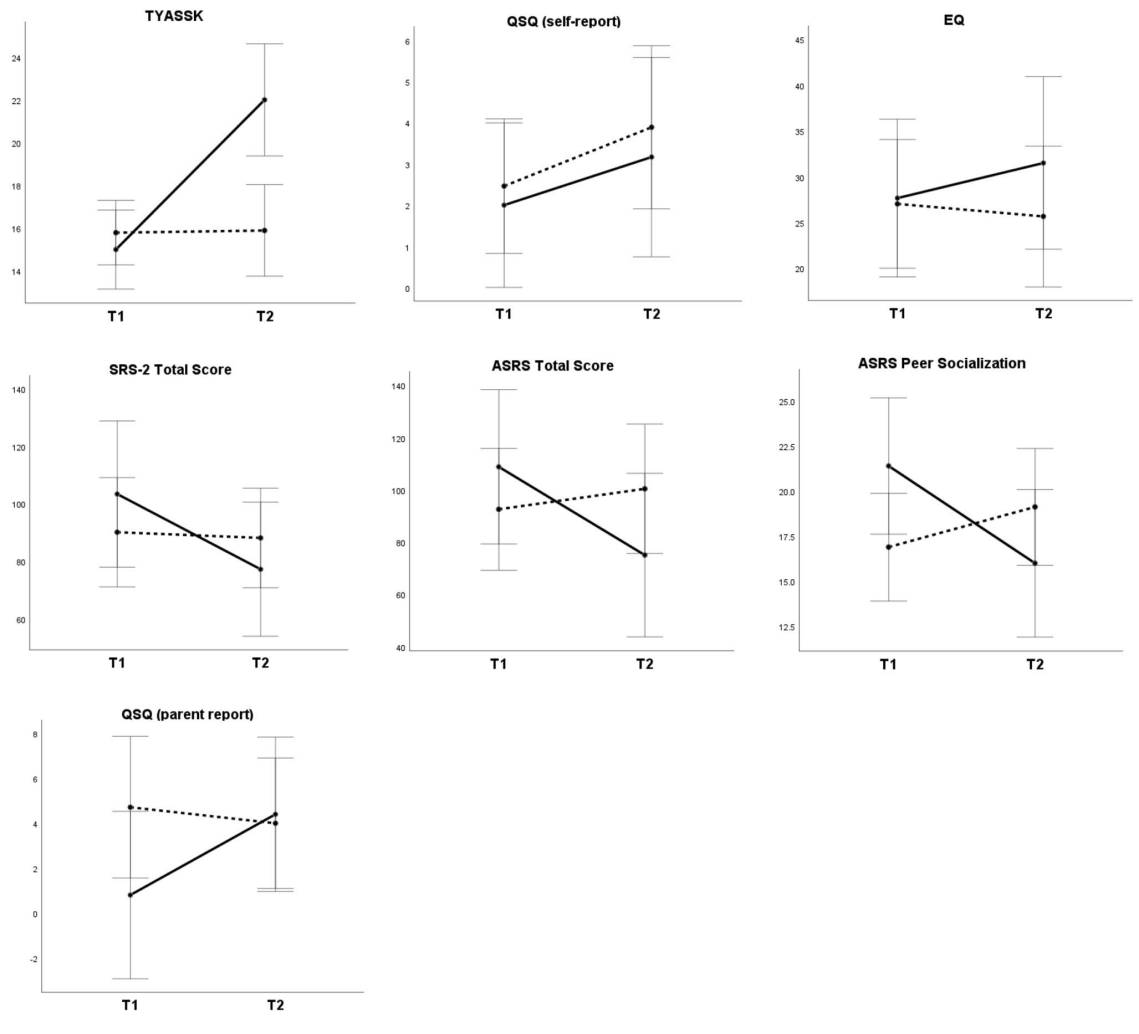
Means and standard errors for outcome variables in the Treatment (solid line) and Waitlist Control Group (dotted line) at Pre-test (T1) and Post-test (T2). TYASSK = Test of Young Adult Social Skills Knowledge; QSQ = Quality of Socialization Questionnaire; SRS-2 = Social Responsiveness Scale-2; ASRS = Autism Spectrum Rating Scale; QSQ = Quality of Socialization Questionnaire; EQ = Empathy Quotient

### Ecological Validity

There were no significant differences in treatment satisfaction between TG and WCG, so the results will be described on the whole-group level, but the group-specific data can be found in Table S2 and Table S3 in the Appendix.

Overall, young adults rated the PEERS^®^ program as helpful. They reported learning how to establish and maintain relationships with others better (*M* = 5.57; *SD* = 0.94; from 1 – ‘definitely not’ to 7 – ‘definitely yes’). They would also recommend the program to other autistic people (*M* = 6.57; *SD* = 0.76; from 1 – ‘definitely not’ to 7 – ‘definitely yes’). Specific components of the program also gained positive feedback, with ‘social coaches support’ eliciting the best ratings (*M* = 6.07; *SD* = 1.44). Young adults assessed their time burden to participate in the program as low-to-moderate (*M* = 2.86; *SD* = 1.41; from 1 – ‘little burden’ to 7 – ‘too much burden’).

With regard to open-ended questions, young adults responses underscored the positive attitude of group leaders (*n* = 3), good atmosphere during the classes (*n* = 2), as well as helpfulness of behavioral exercises (*n* = 2), role-play demonstrations (*n* = 2), homework assignments (*n* = 2), and support of social coaches (*n* = 2). Most participants would not change anything in the program (*n* = 5). Others suggested lengthening the sessions’ duration, adding more content related to dating, providing more opportunities for in-group socialization, and more assistance in seeking extracurricular activities (as potential sources of friends).

Social coaches viewed the program as helpful in teaching young adults how to establish and maintain relationships (*M* = 5.38; *SD* = 1.50) and would strongly recommend the program to other people (*M* = 6.69; *SD* = 0.63). All the program’s main components – homework review, didactic lessons, and role-play videos – gained high satisfaction scores (all *Ms* > 5.50). Social coaches rated the workload of the program as moderate for young adults (*M* = 3.69; *SD* = 2.10 from 1 – ‘little burden’ to 7 – ‘too much burden’) and for themselves (*M* = 3.85; *SD* = 1.63). There were no significant differences in treatment satisfaction between parents and peers as social coaches (see Table S3 for detailed results in these subgroups). However, parents tended to rate the program helpfulness slightly better and their time burden as smaller than peer coaches did.

In their open comments, social coaches appreciated group leaders’ professionalism and commitment (*n* = 6), opportunity to share experiences and learn from each other (*n* = 5), individualized approach and feedback (*n* = 3), receiving handouts and role-play videos to watch at home (*n* = 2), concrete steps and rules to follow (*n* = 2). Moreover, social coaches suggested lengthening the program or extending the sessions to two hours (*n* = 4), organizing booster sessions for participants once a year (*n* = 1), spending more time on reading non-verbal cues (*n* = 1), and adjusting some homework assignments when a social coach does not live with a participant (*n* = 1).

Peer coaches (psychology students) reported that during the program they learned a lot about autistic adults and their perspectives (*n* = 4), improved their own communication skills (*n* = 2), developed their patience (*n* = 1), and were inspired for future career development (*n* = 1).

### Maintenance of the Treatment Effects

Young adults and their parents were contacted six months after the treatment’s conclusion to examine the maintenance of the intervention effects. In TG, five young adults and four parents responded (83.3% and 80.0% response rate, respectively). In the WCG, six young adults and five parents responded (75.0% and 62.5% response rate, respectively). There were no differences in any demographic or outcome measures at T1 between those who completed the follow-up and those who did not. To avoid decreasing the statistical power of the analyses, maintenance of the treatment effects was calculated on a whole-group level using within-subjects ANOVAs (pre-test vs. post-test vs. follow-up). Results indicated statistically significant effects of Time for knowledge about social skills (TYASSK), empathy (EQ), parent-reported difficulties related to autism (SRS-2 and ASRS), and parent-reported problems in peer relations (ASRS Peer Socialization), all with large effect sizes (partial η^2^ = 0.359-0.838). The number of get-togethers (QSQ) was not significant either for parent or self-reports.

**Fig. 3:**
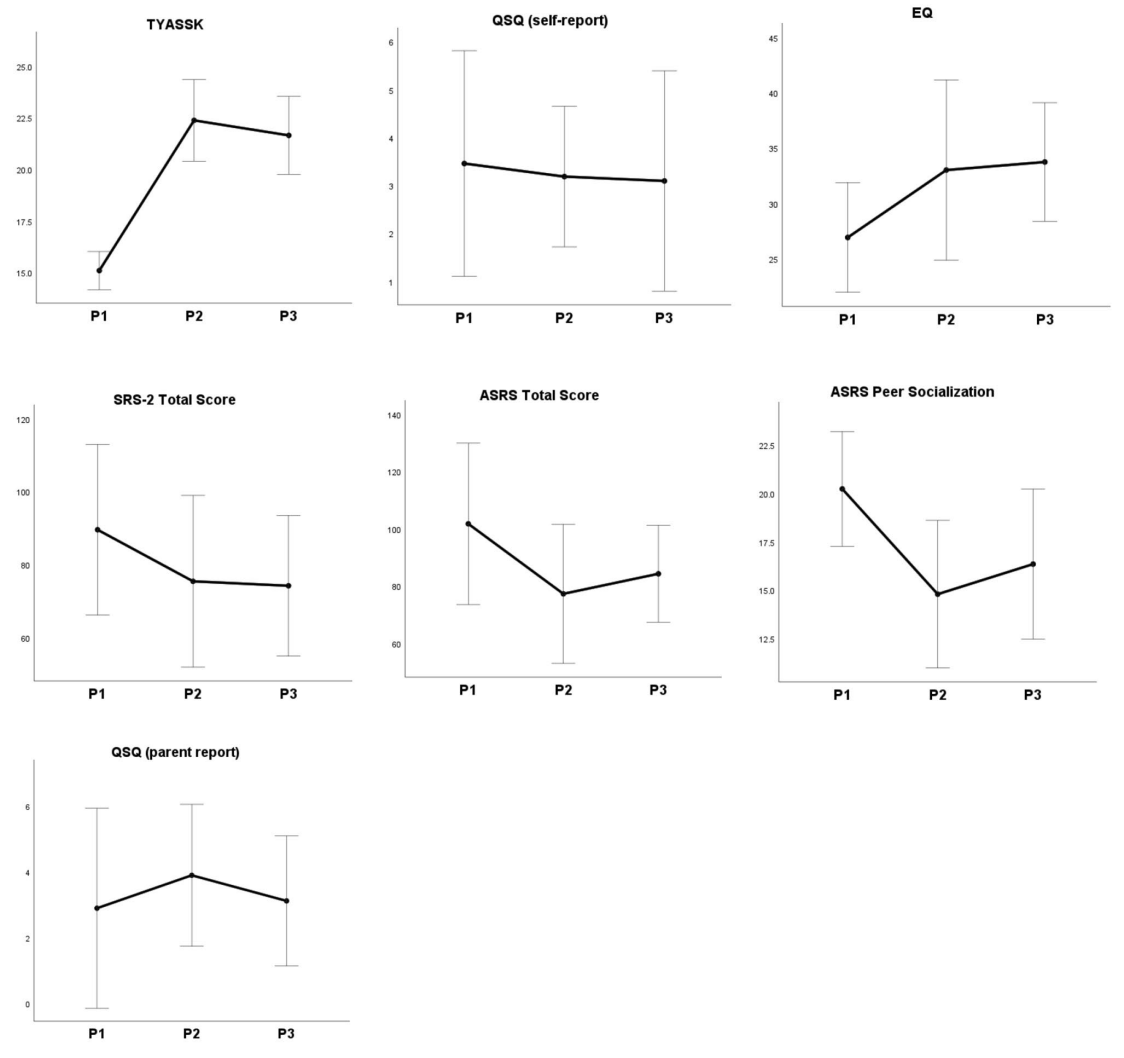
Means and standard errors for outcome variables at Pre-test (P1), Post-test (P2), and Follow-up (P3), Treatment Group and Waitlist Control Group combined. TYASSK = Test of Young Adult Social Skills Knowledge; QSQ = Quality of Socialization Questionnaire; SRS-2 = Social Responsiveness Scale-2; ASRS = Autism Spectrum Rating Scale; QSQ = Quality of Socialization Questionnaire; EQ = Empathy Quotient

As shown in Table 3; Fig. 3, posthoc comparisons indicated maintenance of most of the treatment results. For all the effects reported above, there were statistically significant changes from pre-test to post-test, as well as from pre-test to follow-up in the expected directions, and no differences between post-test and follow-up. The exception was a difference between pre-test and follow-up in ASRS Total Score that did not reach significance (*p* = .12). All the effects remained significant after adjusting for the number of comparisons.

**Table 3:** Maintenance of treatment effects (whole-group analyses; *n* = 11)

	Pre (P1)		Post (P2)		Follow-up (P3)		P2-P1			P3-P2			P3-P1	
	*M (SD)*		*M (SD)*		*M (SD)*		*M*_*d*_ *(SE)*	*p*		*M*_*d*_ *(SE)*	*p*		*M*_*d*_ *(SE)*	*p*
*Young Adult report*														
TYASSK	15.09 (1.36)		22.36 (2.94)		21.64 (2.80)		7.27 (0.86)	< 0.001*		-0.73 (0.65)	0.29		6.55 (0.84)	< 0.001*
QSQ Total get-togethers	3.45 (3.50)		3.18 (2.18)		3.09 (3.42)		0.27 (0.69)	0.70		-0.09 (0.86)	0.92		-0.36 (0.87)	0.68
EQ	26.91 (7.38)		33.00 (12.12)		33.73 (7.98)		6.09 (2.39)	0.03*		0.73 (2.37)	0.77		6.82 (1.91)	0.01*
*Parent report*														
SRS-2 Total Score^a^	89.56 (30.38)		75.44 (30.60)		74.22 (25.06)		-14.11 (5.56)	0.03*		-1.22 (4.64)	0.80		-15.33 (6.39)	0.04*
ASRS Total Score^a^	101.67 (36.64)		77.22 (31.60)		84.22 (20.04)		-24.44 (6.48)	0.01*		7.00 (7.36)	0.37		-17.44 (10.16)	0.12
ASRS Peer Socialization^a^	20.22 (3.87)		14.78 (4.97)		16.33 (5.05)		-5.44 (1.21)	0.002*		1.56 (1.86)	0.43		-3.89 (1.25)	0.015*
QSQ Total get-togethers^a^	2.89 (3.95)		3.89 (2.80)		3.11 (2.57)		-0.78 (1.30)	0.57		-1.44 (1.36)	0.32		0.22 (0.91)	0.81

Note. TYASSK = Test of Young Adult Social Skills Knowledge; QSQ = Quality of Socialization Questionnaire; SRS-2 = Social Responsiveness Scale-2; ASRS = Autism Spectrum Rating Scale; QSQ = Quality of Socialization Questionnaire; EQ = Empathy Quotient

^a^ n = 9

* Statistically significant under Benjamini-Hochberg procedure (FDR = 0.10).

### Impact of Treatment Modality

In order to explore potential differences between hybrid (received by TG) and in-person delivery mode (received by WCG) of the intervention, mixed ANOVAs were conducted with Group as a between-subjects factor (TG vs. WCG) and Time as a within-subjects factor (active intervention periods for each group: T1 vs. T2 for TG; T2 vs. T3 for WCG).

There were no significant interaction effects for any self-report variables: knowledge about social skills (TYASSK: *F* (1, 12) = 0.07; *p* = .795; partial η^2^ = 0.006), number of get-togethers (QSQ Young Adult: *F* (1, 12) = 1.06; *p* = .325; partial η^2^ = 0.081), and empathy (EQ: *F* (1, 12) = 0.25; *p* = .627; partial η^2^ = 0.020). Initially, two parent-reported measures of autism-related difficulties showed significant interaction effects: SRS-2 Total Score (*F* (1, 11) = 5.44; *p* = .040; partial η^2^ = 0.331) and ASRS Total Score *F* (1, 11) = 5.18; *p* = .044; partial η^2^ = 0.320. However, these effects did not remain significant after applying a correction for multiple comparisons using the Benjamini-Hochberg procedure. Finally, analyses showed no interaction effects in parent-reported peer problems (ASRS Peer Socialization: *F* (1, 11) = 0.73; *p* = .411; partial η^2^ = 0.062) and the number of get-togethers, in case of which a non-parametric test on difference scores was conducted (QSQ Parent: *U* = 13.5; *Z* = -0.96; exact *p* = .354; Cohen’s *r* = .27).

In line with previous results, analyses showed statistically significant main effects of Time but not Group for all the outcome variables, except for the number of get-togethers (QSQ Young Adult and QSQ Parents), in which no significant main effects were found.

## Discussion

The goal of the study was to establish the evidence base for the Polish adaptation of the PEERS^®^ for Young Adults curriculum. Results indicated that participation in the program leads to significant improvements in young adults’ social knowledge and social skills, diminishing peer-related problems, but limited gains in social engagement. Importantly, the intervention effects were maintained over time and the curriculum was highly accepted by autistic young adults, their parents, and peers involved as social coaches.

In line with the original RCTs of the PEERS^®^ curriculum from the United States (Gantman et al., 2012; Laugeson et al., 2015), the Polish adapted version of the program showed positive effects on different aspects of young adults’ social functioning. A Total Score and four out of five treatment subscales of the SRS-2 showed significant time by group interactions, including Social Cognition, Social Communication, Social Motivation, and Restricted and Repetitive Behaviors. Social communication is closely related to social skills taught in the program, such as holding a two-way conversation, and social cognition is fostered via perspective-taking questions during role-play demonstrations. Encouragingly, a change in the Social Motivation subscale indicates that young adults increased also their motivation to engage in social interactions, perhaps as a result of growing self-competence and positive experiences during get-togethers with peers. However, the Social Awareness subscale did not show an improvement, corroborating one of the social coaches’ suggestions that reading non-verbal cues may require more time in the curriculum.

These results are further validated by the second measure of difficulties related to autism spectrum – ASRS. The Treatment Group showed a large improvement both in a Total Score and a Peer Socialization subscale that related specifically to difficulties in peer interactions. Notably, in the Waitlist Control Group parents reported an increase in autism-related difficulties, both for the Total Score and the Peer Socialization subscale. This might stem from the difficult situation during the COVID-19 pandemic with limited opportunities for both socialization with peers and therapeutic support, which added to amplified anxiety and stress (Oomen et al., 2021). In light of this study, group-based social skills interventions may help prevent the negative impact of social isolation among autistic young adults.

Parent report is consistent with young adults’ report of social cognition and related social skills, measured by the EQ. Interestingly, the EQ showed a significant effect only in a follow-up, consistent with results obtained by Laugeson and colleagues (2015). This may indicate that young adults need more time to consolidate new skills and relate them to their self-perception. Moreover, young adults improved their knowledge about social skills, as measured by TYASSK.

The treatment did not show consistent improvement in young adults’ social engagement. After an initial increase in the number of get-togethers with peers (significant only by parent report), in the follow-up, it came back to the previous level. This pattern resembles results obtained in the Korean adaptation of PEERS^®^ for Young Adults (Oh et al., 2021) and in some adaptations of PEERS^®^ for Adolescents, including a Polish adaptation (Płatos et al., 2022). This suggests that there can be factors contributing to social engagement other than social skills, such as social motivation, organizational skills, or availability of socialization opportunities. In particular, this last circumstance could have affected the results, as pandemic-related mobility and gatherings restrictions were in effect for most of the study duration. However, the results can also reflect a discrepancy between social skills acquisition and social performance in real-life situations. Further research should address both the reasons for such a discrepancy and possible solutions, such as post-treatment support focused on social engagement and skills generalization.

Finally, the majority of the young adults did not report going on dates either before or after the treatment. Noteworthy, young adults were not encouraged to go on dates if they did not feel ready or were not interested in anybody romantically. Nonetheless, more direct measures are needed to assess dating skills, and this module of the PEERS^®^ curriculum warrants further investigation.

The current study represents the second cultural adaptation of the PEERS^®^ for Young Adults curriculum and one of the few RCTs examining the efficacy of SST for autistic adults (Dubreucq et al., 2021). The adaptation efforts included updating the curriculum based on multiple sources, recreating some of the program’s materials (including role-play videos), and conducting a pilot of the intervention. One of the most important changes to the original curriculum was to include peers (psychology students) as social coaches when parents were unavailable or unsuitable for the role. Engaging peers to practice social skills with young adults was deemed developmentally appropriate and proved to be feasible. Moreover, qualitative results indicated that being a peer coach increased their autism awareness and communication skills, which represents an additional benefit of the program. However, the sample of peer social coaches was too small and unevenly distributed across the study conditions to fully ascertain the impact of this change on the intervention effects.

The input from autistic people provided important guidance both in the process of the program’s adaptation and during its evaluation. Some of the terms were modified (such as “training”) if they had negative connotations for some members of the autistic community. Importantly, using specific social skills was presented as a personal choice, not a necessity, to discourage potentially harmful masking strategies (Cook et al., 2021). Lastly, the study is also one of the few to provide quantitative and qualitative data on treatment satisfaction (Monahan et al., 2021), showing its ecological validity. The intervention was rated as attractive and not overly burdening by both young adults and social coaches, which was further confirmed by the high attendance rates throughout the program.

The trial was conducted during the COVID-19 pandemic, which had a profound impact on its execution. Participants in the Treatment Group received almost half of the sessions remotely, while those in the Waiting Control Group held all the meetings in person, although with some restrictions on social gatherings still in effect. However, secondary analyses showed that remote instruction did not affect the intervention effects, corroborating recent findings from other studies (Adler et al., 2022; Estabillo et al., 2022). As the importance of online treatment delivery increases, further research should focus on direct comparisons of remote, hybrid, and in-person modes of instruction.

### Limitations

The study has some limitations. First, the sample was small, so some statistical analyses might lack sufficient power to detect the effects. Second, the study lacked a blinded, observational assessment of social skills, which would provide external validation of the self and parent reports. It would be useful to adapt available measures (e.g., Ratto, Turner-Brown, Rupp, Mesibov, & Penn, 2011) but also to develop new ones to examine skills related to, for example, the use of humor, electronic communication, or dating, which are currently difficult to assess. While such measures were not available during the study conception and not possible to be applied during the pandemic, the work on them is currently underway, so they can inform future research. Third, the COVID-19 pandemic forced some changes in the study protocol, including the mode of instruction (partial online delivery) and the assessments (online socialization with peers). This could have had an impact on the intervention effects, especially on young adults’ social engagement. However, an effort was made to ascertain the impact of any changes to the study protocol and to keep the assessment process standardized. Fourth, one-third of participants received group and/or individual psychotherapy that could have addressed social skills or understanding. However, the number of participants receiving such treatment was distributed evenly across the study conditions.

## Conclusion

The study represents one of the first cultural adaptations of the PEERS^®^ for Young Adults curriculum and SST for autistic adults, in general. It provides evidence for the effects of this intervention in improving the social skills, social cognition, and social knowledge of young adults on the autism spectrum, according to self and parent reports. Importantly, these effects were maintained over time, with some additional gains at follow-up. Lastly, the Polish adaptation of PEERS^®^ for Young Adults showed high feasibility and acceptability that should foster its successful implementation.

## Electronic Supplementary Material

Below is the link to the electronic supplementary material.

Supplementary Material 1
