# Supplementary material for: Fostering Friendship and Dating Skills Among Adults on the Autism Spectrum: A Randomized Controlled Trial of the Polish Version of the PEERS® for Young Adults Curriculum
Source: J Autism Dev Disord. 2023 Apr 12;54(6):2224–39. doi: 10.1007/s10803-023-05921-y (PMC10090714; doi:10.1007/s10803-023-05921-y)
Supplement: Supplementary file 1 — Supplementary Material 1 [file 10803_2023_5921_MOESM1_ESM.docx]

Appendix

Table S1. Overview of the Polish adaptation of the PEERS® for Young Adults curriculum

| Session | Didactic lesson | Description | Cultural adaptations and updates |
| --- | --- | --- | --- |
| 1 | Trading Information and Starting Conversations | Overview of the program.  Characteristics and types of peer relationships.  Rules for trading information.  Steps for starting individual conversation. | Following materials from the PEERS Certified Provider Training, an online friend was added to the types of relationships discussed.  Some language considerations specific to Polish were added, e.g., a word ‘friend’ is reserved only for very close relationships, a word ‘colleague’ can mean both classmate and someone we like and spend time with.  The game ‘Jeopardy’ was adapted to the type of quizzes that Polish young adults know. |
| 2 | Trading Information and Maintaining Conversations | Rules for having two-way conversation, including asking open-ended and follow-up questions and non-verbal communication.  Common topics of conversations with peers. | Common conversational topics among young adults were adapted to the Polish context.  Following the Telehealth version of the PEERS curriculum, new rules were added: Don’t be brutally honest; don’t interrupt. |
| 3 | Finding a Source of Friends | Brainstorming and identifying appropriate peer groups (crowds) young adults belong to or would like to.  Cues for acceptance or rejection in peer relationships. | Modification of the crowds popular among young adults: some of the original crowds were not relevant in Poland (e.g., preppies, fraternities/sororities, surfers) and some new were introduced (e.g., animal lovers, environmental activists, vegan/vegetarian groups). |
| 4 | Electronic Communication | Steps for exchanging contact information and for starting and ending a phone call.  Rules for using the Internet.  Rules for using social media. | Leaving a voicemail message is not common among young adults in Poland anymore; instead, steps for sending a text message if someone did not answer the phone were introduced.  Types of social media were updated for those popular among Polish young adults (Instagram, Facebook, Reddit, LinkedIn).  Following the Telehealth version of the curriculum, new rules for using the Internet and social media were added. |
| 5 | Appropriate Use of Humor | Basic rules for appropriate use of humor.  Paying attention to humor feedback.  Determining one’s own attitude towards joking. | Knock-knock jokes were replaced by jokes used by young adults in Poland. |
| 6 | Entering Group Conversations | Steps for joining group conversation. | None. |
| 7 | Existing Conversations | Steps for exiting the conversation when accepted, initially accepted but then rejected, or rejected. | None. |
| 8 | Get-togethers | Rules and steps for planning, preparing, starting, and ending a successful get-together, as well as appropriate behaviors when meeting with friends. | Modifications in starting a get-together held at home: a step ‘Show them around’ was omitted as it is not common for Polish young adults, and a step ‘Take their jacket or coat’ was introduced as it is regularly practiced. |
| 9 | Dating Etiquette: Letting Someone Know You Like Them | Rules and steps for showing someone romantic interest, including flirting, giving compliments, talking to mutual friends, and appropriate asking if they are dating someone. | None. |
| 10 | Dating Etiquette: Asking Someone on a Date | Rules and steps for asking somebody on a date, accepting rejection, and turning someone down. | None. |
| 11 | Dating Etiquette: Going on Dates | Rules for having successful dates, including planning and preparation for a date, safety considerations, as well as appropriate beginning and ending a date. | A rule ‘Drive yourself to and from the date’ was modified to ‘Arrange transportation to and from your date,’ as driving a car is not very common in young adults on the autism spectrum in Poland.  Role-play demonstrations and videos for ‘Beginning a date’ and ‘Ending a date’ were set in a cafeteria, as it is not common for young adults to have one of the first dates at home. |
| 12 | Dating Etiquette: Dating Do’s and Don’ts | General rules for dating and steps for handling sexual pressure from partners. | A rule ‘Don’t disclose your diagnosis at first’ was extended into a discussion about when and how to disclose an autism spectrum diagnosis. Consequences of different choices are discussed and the importance of the individual decision related to one’s identity is emphasized. |
| 13 | Handling Disagreements | Rules and steps for responding and bringing up disagreements. | None. |
| 14 | Handling Direct Bullying | Instructions for appropriate responding to teasing and embarrassing feedback.  Strategies for dealing with physical bullying (e.g., hitting, forcing to do something, taking things). | Teasing comebacks were adapted so they reflect ecologically valid responses to teasing among Polish young adults. |
| 15 | Handling Indirect Bullying | Strategies for handling bullying on the internet, including social media.  Strategies for handling gossips, including spreading the rumor about oneself. | None. |
| 16 | Moving Forward and Graduation | Summary of the program and instructions for maintenance of effects and further development.  Graduation party – social time with board games and food and beverages. | A new rule was introduced: ‘Be yourself’ to encourage participants to consider what is important to them and avoid potentially harmful masking strategies. |

Table S2. Young adult’ satisfaction of the PEERS® program

|  | | Treatment Group  (*n* = 6) | | Waitlist Control Group (*n* = 8) | | Summary (*n* = 14) |
| --- | --- | --- | --- | --- | --- | --- |
|  | | *M (SD)* | | *M (SD)* | | *M (SD)* |
| *Young adults’ satisfaction* | |  |  |  |  |  |
| How helpful were the following components  of the PEERS program for you:  (1 - not helpful at all; 7 - very helpful) | |  |  |  |  |  |
|  | Group discussion and role-play demonstrations | 6.00 (1.26) |  | 5.88 (1.46) |  | 5.93 (1.33) |
|  | Behavioral exercises performed  during classes | 5.83 (1.17) |  | 5.75 (1.39) |  | 5.79 (1.05) |
|  | Homework assignments | 5.33 (1.51) |  | 5.75 (1.39) |  | 5.57 (1.40) |
|  | Social coach support | 5.67 (1.51) |  | 6.38 (1.41) |  | 6.07 (1.44) |
| How do you rate the time burden to participate in the program?  (1 – little burden; 7 – too much burden) | | 3.0 (1.10) |  | 2.75 (1.67) |  | 2.86 (1.41) |
| Do you think that by participating in the program you have learned how to establish and maintain relationships with others better?  (1 – definitely not; 7 – definitely yes) | | 6.17 (0.98) |  | 5.13 (0.64) |  | 5.57 (0.94) |
| Would you recommend this program to other people on the autism spectrum?  (1 – definitely not; 7 – definitely yes) | | 6.33 (0.82) |  | 6.75 (0.71) |  | 6.57 (0.76) |

Table S3. Social coaches’ satisfaction of the PEERS® program

|  | | Treatment Group – parents as social coaches^a^ (*n* = 5) | | Waitlist Control Group – parents as social coaches (*n* = 2) | | Waitlist Control Group – peers as social coaches (*n* = 6) | | Summary (*n* = 13) |
| --- | --- | --- | --- | --- | --- | --- | --- | --- |
|  | | *M (SD)* | | *M (SD)* | | *M (SD)* | | *M (SD)* |
| How helpful were the following components of PEERS for you:  (scale 1 - not helpful at all; 7 - very helpful) | |  |  |  |  |  |  |  |
|  | Homework review during classes | 6.80 (0.45) |  | 7.00 (0.00) |  | 5.50 (1.05) |  | 6.23 (1.01) |
|  | Didactic lessons and watching role-play videos during classes | 6.80 (0.45) |  | 7.00 (0.00) |  | 5.33 (1.37) |  | 6.15 (1.21) |
|  | Opportunity to watch role-play videos at home | 6.80 (0.45) |  | 7.00 (0.00) |  | 4.00 (1.79) |  | 5.54 (1.90) |
| How do you rate your time burden to participate in the program?  (1 – little burden; 7 – too much burden) | | 4.00 (1.87) |  | 2.50 (2.12) |  | 4.17 (1.33) |  | 3.85 (1.63) |
| How would you rate the time burden for the person you supported to participate in the program?  (1 – little burden; 7 – too much burden) | | 2.40 (1.95) |  | 2.50 (2.12) |  | 5.17 (1.33) |  | 3.69 (2.10) |
| Do you think that by participating in the program the person you supported has learned how to establish and maintain relationships with others better?  (1 – definitely not; 7 – definitely yes) | | 6.20 (0.45) |  | 6.50 (0.71) |  | 4.33 (1.63) |  | 5.38 (1.50) |
| Would you recommend this program to other parents of young adults on the autism spectrum / other people?  (1 – definitely not; 7 – definitely yes) | | 7.00 (0.00) |  | 7.00 (0.00) |  | 6.33 (0.82) |  | 6.69 (0.63) |

Note. ^a^ TG included only one peer coach who did not report on the satisfaction due to organizational reasons.
